# Matrices for Sensors from Inorganic, Organic, and Biological Nanocomposites

**DOI:** 10.3390/ma4081483

**Published:** 2011-08-24

**Authors:** Claudio Nicolini, Victor Sivozhelezov, Valter Bavastrello, Tercio Bezzerra, Dora Scudieri, Rosanna Spera, Eugenia Pechkova

**Affiliations:** 1 Nanoworld Institute Fondazione ELBA Nicolini, Bergamo 24100, Italy; E-Mails: vsivo@ibf.unige.it (V.S.); rspera@ibf.unige.it (R.S); epechkova@ibf.unige.it (E.P.); 2 Chair of Biophysics, University of Genova, Genova 16121-16167, Italy; E-Mails: vbavastrello@ibf.unige.it (V.B.); tbezzerra@ibf.unige.it (T.B.); dscudieri@ibf.unige.it (D.S.)

**Keywords:** nanocomposite, matrix, sensors

## Abstract

Matrices and sensors resulting from inorganic, organic and biological nanocomposites are presented in this overview. The term nanocomposite designates a solid combination of a matrix and of nanodimensional phases differing in properties from the matrix due to dissimilarities in structure and chemistry. The nanoocomposites chosen for a wide variety of health and environment sensors consist of Anodic Porous Allumina and P450scc, Carbon nanotubes and Conductive Polymers, Langmuir Blodgett Films of Lipases, Laccases, Cytochromes and Rhodopsins, Three-dimensional Nanoporous Materials and Nucleic Acid Programmable Protein Arrays.

## 1. Introduction

Nanocomposite matrices are based on the combination of inorganic, organic and biological (such as proteins and DNA) materials and as such they constitute a useful construct for a wide range of applications for health and environment, including microarrays and sensors [1]. For sensor application of nanomaterials, see reviews [2,3]. The latter is the main objective of this overview. The rationale behind it is that nanocomposite is the natural state of most biomolecules. Indeed, *in vivo*, proteins are nanostructured together with other phases such as lipids, rather than free in solvent/cytosol. The term “biological nanocomposite” is used in the literature for the most obvious cases of wood and bone, but in fact most biological materials are approaching the definition of nanocomposites. Most importantly, biomembranes, unlike e.g., lipid bilayers, are not monophasic in their composition. Apart from lipids, they contain integral membrane proteins naturally nanostructured in such a manner as to perform their functions such as ion channels or respiratory (redox) systems. Another example of a biological nanocomposite is the cellular nucleus which is arranged in periodical repeats of nucleosomes, which in turn are nanocomposites with superhelical DNA forming a matrix by winding onto special proteins called histones. Similarly, the intracellular medium is not a material nanostructured by cytoskeleton, which is again not monophasic at the nanometer scale. For sensor applications, the rationale for usage of nanocomposites is based on the fact that nanocomposite matrix is in a way the ideal immobilization medium for sensory molecules. Only if the matrix is nanostructured, is it possible to control position and orientation of sensory molecules immobilized in the matrix. Then, the response of sensory molecules to the expected stimulus becomes, first, uniform within the sensor, and second, controllable by chemical modification (often called functionalization) as in the case of organic/inorganic matrices, or site-directed mutagenesis as in biological matrices.

This overview describes the utilization of a wide range of complex nanocomposites for optimal sensor implementation, either inorganic (such as anodic porous alumina) with cytochromes P450, organic (such as carbon nanotubes with conducting polymers), and biological (such as Nucleic Acid Programmable Protein Arrays or LB multilayers of proteins of primary interest, *i.e*., lipases, rhodopsins, laccases and heme metalloproteins). Special attention is paid to detecting substances significant for either environment or medicine by means of a wide variety of detection methods, and to the resulting nanosensors being built over the years up to the most recent under construction.

Only a few proteins were included with respect to biosensors since we aim to present an overview with emphasis in our own work. We do not attempt here to describe all nanocomposite based biosensors and therefore the list of proteins covered by this overview is far from complete. We include a few representative examples and therefore we chose proteins with outstanding biosensor and/or enzymatic properties. Specifically, we describe Cytochrome P450scc for cholesterol sensor to show that the anodic porous alumina technique is perfectly compatible with cytochromes P450. Enzymes of the latter class can detect not only cholesterol but a wide range of organic substances because a wide variety of cytochromes P450 is encoded by mammalian genomes. In fact, the Cytochrome P450 able to metabolize a xenobiotic can start its expression in an organism once the organism becomes exposed to the given xenobiotic. Rhodopsins and bacterial globins were picked because they are naturally occurring light and oxygen sensors, respectively. Lipases were picked because they are present in very diverse living organisms from bacteria to mammalians, and have as their substrates a wide range of fatty acids and triglycerides. Finally, laccases are extracellular enzymes able to function in a wide variety of conditions. To summarize, among the proteins available for biosensor development, these proteins are the most diverse functionally. They are also very diverse structurally, *i.e.*, in terms of arrangements of their alpha helices and/or beta sheets. This allows the assumption that the set or proteins presented herein is representative, although not exhaustive. In addition, this review is complementary in the choice of biomolecules to the recent review focusing on nanostructures in biosensing [4].

## 2. Anodic Porous Allumina and P450scc for Cholesterol Sensor

**Figure 1:**
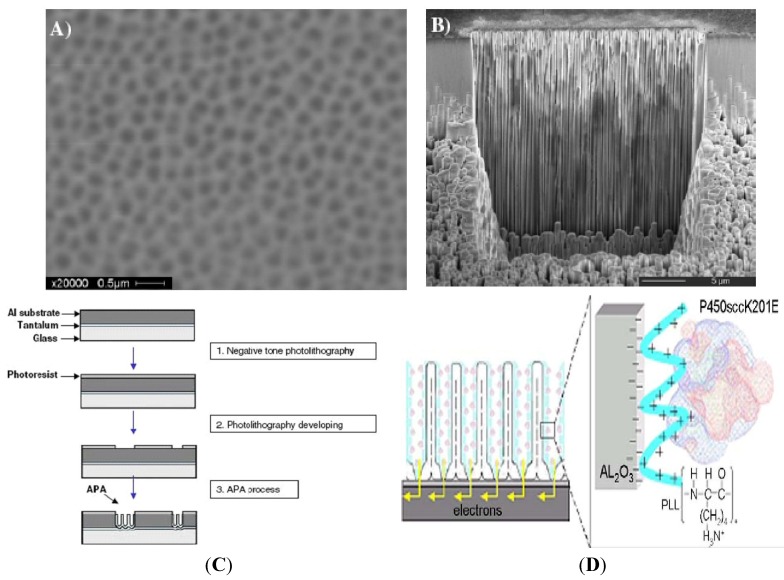
(**A**) SEM picture (top view) showing highly ordered anodic porous alumina on the surface of the microarray spot, resulting at the end of the photolithographic microstructuring technique and the two-step anodization process; (**B**) FIB (focused ion beam) system images of cross-sectional morphologies of the microarray spot, resulting at the end of the photolithographicmicrostructuring technique and the two-step anodization process. FIB-FEI® (www.fei.com) —measurements were made with gallium ions—37 pA—both for cutting and imaging; (**C**) Schematic diagram of anodic porous alumina (APA) microstructuring process; (**D**) APA functionalized rhodium–graphite s.p.e. working electrode. Schematic view illustrating the direct electron transfer between the cytochrome P450scc catalytic “core” and the APA modified working electrode. In the box is shown the specific interaction between the cytochrome P450scc negative surface (blue) and the positive charges of poly-l-lysine.

Anodic porous alumina substrate was reported for applications in biomedical diagnostics ([5,6] and references therein). A photolithographic microstructuring technique (C) for an ordered nanopore array fabrication is reported in Figure 1, along with the typical Anodic Porous Allumina (A,B) obtained and the working electrode (D) being built for a cholesterol sensor based on the anchored P450scc cytochrome.

The process uses a negative resist with hydrophobic properties increasing specificity to biomolecule linking. Nanoporous alumina is formed by an anodic process and yields straight holes with high aspect ratio: its use as substrates for DNA-microarray or protein-chip application offers several advantages over conventional supports, making them very attractive to use as supports for biological sample microarray application. Figure 1A shows a typical sample obtained by using the photolithographic microstructuring process described above; microarrays with spots 120 μm wide, 20–25 μm deep and 100 μm spaced out were prepared. Each spot appears to be made of hexagonally ordered pore domains prepared by a selforganization process under specific anodization conditions (Figure 1A). This nanopatterning technique leads to a sharp edge. The anisotropy of the process can be seen with the FIB system. The side walls of the structures are very steep, and their roughness is determined by the quality of the mask (Figure 1B).

In particular, anodic porous alumina (APA) was used to improve the mechanical stability of electrodes based on P450scc for LDL-cholesterol detection and measurement [5]. Cytochromes P450 are discussed in detail in subsequent sections of the review. Therefore the inorganic matrix was used for immobilization of P450scc macromolecules preserving their electronic sensitivity to its native substrate, cholesterol (Figure 1C). Moreover with cavities implemented in this work 275 nm wide and 160 microm deep (as demonstrated with AFM and SEM measurement) can be tuned to optimize immobilization of other enzymes in diameter modifying the synthesis parameters.

**Figure 2:**
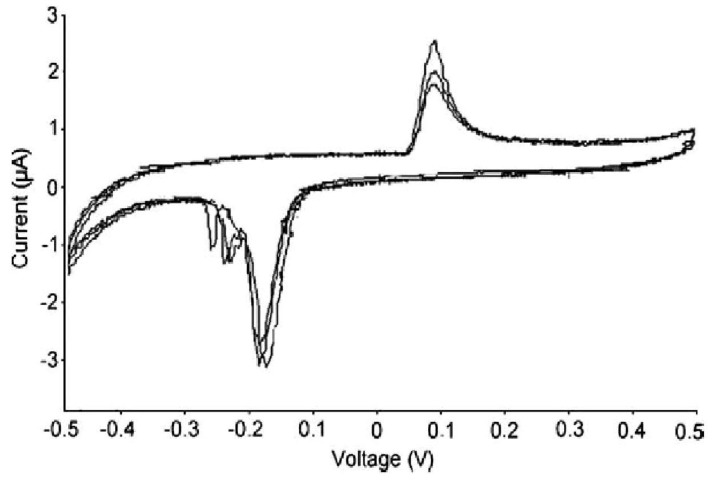
Cyclic voltammograms (CV), showing the I–V current-voltage curves of s.p.e. of APA–P450scc Electrode in presence of substrate LDL-cholesterol. The s.p.e. of APA–P450scc electrode was investigated, after a month, in a 10 mM K-phosphate buffer pH 7.4 in presence of LDL-cholesterol. (**a**) P450; (**b**) LDL 0.5 mg/mL; (**c**) LDL1.1 mg/mL; and (**d**) LDL1.6 mg/ml. Results are representative of one of three similar experiments.

Figure 1D shows a schematic model of the interaction between APA modified electrode and P450scc cytochrome. An organic layer of the poly-cathion PLL was deposited between APA nanopores and the enzyme. The PLL provides both a chemically molecular anchorage and a homogenous and direct electron transfer (Figure 2).

AFM analysis of the APA membrane shows set onto the working electrode. Considering the pore diameter (275 nm), the P45scc longest dimension and the PLL’s layer thickness, and also considering the not-excessive concentration of both solutions, the APA matrix is yet clearly visible and its surface is barely changed, as a result of the deposition of the above two elements. Moreover, the ordered structure of the APA film shows ordered pores arranged in hexagonal domains (about five pores in 1 µm^2^). To characterize electroactive species, in our case Cytochrome P450scc, on the electrode surface, cyclic voltammetry was used. The current flow through of the working electrode coated with APA/PLL/P450 was evaluated in 10 mMK-phosphate pH 7.4 at scan rate 20 m Vs^−1^. With the exception of a minor side reaction or a contaminant causing a minor redox pair at 0.1−0.25 V, results in Figure 2 shows that there are no redox peaks at the APA/PLL-modified electrode (curve a), while a well-defined and nearly symmetrical redox peak is observed after P450 immobilization. So the redox peak is clearly attributed to electrochemical reaction of the heme-redox-active metal complex of P450. Moreover, the long-term stability of the APA/PLL/P450 covered electrode was investigated, for a period of 30 days at 4 °C. Only a slight change is observed in the current peak. The good stability may be attributed to the enzyme trapped strongly in the APA/PLL film that is stable in the natural medium. It is known that redox potential is strongly dependent on the dielectric surroundings of the redox site; since P450scc is a membrane protein, it is always surrounded by some lipids, and even after treatment with detergents a significant amount of them is still present. After a long time, lipid surroundings undergo rearrangement, and when this happens LDL interacts also with the lipids, resulting in a shift of the redox potential that can be observed after months of shelf life.

Finally, the validity of P450scc immobilization onto APA modified-electrode is confirmed by an analytic comparison among three different enzyme depositions carried out by Nicolini *et al*., Langmuir Schafer (LS), gold nanoparticles and gel matrix [6,7]. The comparison shows that APA matrix allows realization of a more stable, in the time and at the high temperature, modified electrode. Moreover, the range of substrate detectability for APA/PLL/P450scc covered-electrode is greater than the levels measured by our previous P450 immobilization methods.

Thus, we evaluated the functionality of a rhodium–graphite screen-printed electrode modified with anodic porous alumina, and the reported results prove the achievement of: the optimal immobilization of cytochrome P450scc onto the modified electrode in both vertical and horizontal position, achieving far better results with respect to previously ones obtained in our laboratories [6], as well as the optimization of the electron transfer’s stability leading to the optimal detection of LDL cholesterol in the clinical range concentration for longer times of use [8].

In conclusion, the APA-P450scc electrode represents a promising alternative for the existing amperometric biosensor, quite more stable and independent from its working position, either vertical or horizontal.

## 3. Carbon Nanotubes and Conductive Polymers for Gas Sensors

Conducting polymers and carbon nanotubes (CNTs) have recently gained great interest due to their unique chemical and physical properties [9,10,11,12,13,14]. Among their possible interesting applications, these materials can be used in the fabrication of nanocomposites by either embedding little quantity of CNTs inside the polymer matrix of conducting polymers, or depositing thin films of conducting polymers on an array of CNTs. The first method (Table 1) is very simple and is carried out by polymerizing the monomer in the presence of a dispersion of CNTs under controlled conditions by means of either oxidative polymerization or *in situ* electropolymerization, while the second is performed by casting solutions of polymers on CNTs previously synthesized on suitable substrates [15,16,17,18]. The embedding of CNTs in the polymer matrix as well as the deposition of films on CNTs substrates usually lead to the formation of materials showing enhanced chemical and physical properties. These improvements can be important for the fabrication of sensor devices.

**Table 1:** Amounts of reagents used for the synthesis of the materials carried out in 200 mL of 1 M HCl aqueous solution.

Synthesized Material	MWNTs (mg)	SWNTs (mg)	Monomer (g)	Oxidant (g)
POTO	~	~	9.97	5.26
POTO-MWNTs	100.8	~	10.08	5.32
POTO-SWNTs	~	100.5	10.05	5.30
POAS	~	~	10.03	4.61
POAS-MWNTs	99.2	~	9.92	4.56
POAS-SWNTs	~	100.7	10.07	4.63
PDMA	~	~	9.89	4.62
PDMA-MWNTs	99.5	~	9.95	4.65
PDMA-SWNTs	~	100.9	10.09	4.71
PDOA	~	~	10.04	3.70
PDOA-MWNTs	100.2	~	10.02	3.71
PDOA-SWNTs	~	99.7	9.97	3.69

**Figure 3:**
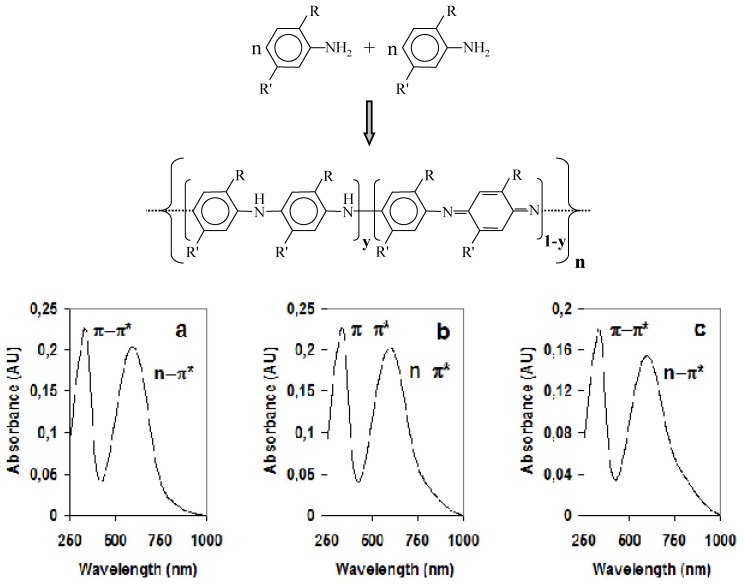

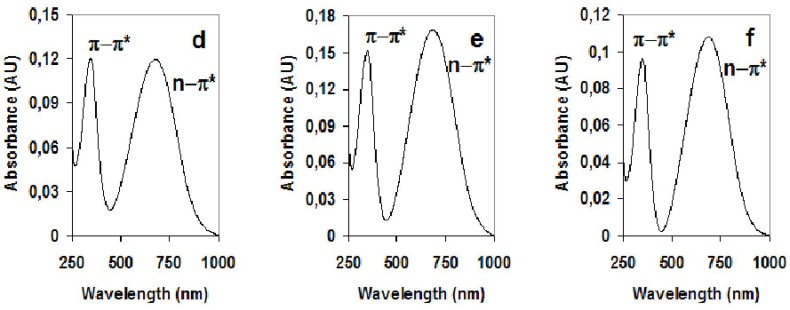
Above: Scheme of the synthesis leading to the formation of a repeat unit starting from monomers. Based on the fraction of imine nitrogen groups per repeat unit, it is possible to obtain different reduced/oxidized polymer chains as follows: Fully reduced form (leucoemeraldine base) for y = 1; fully oxidized form (pernigraniline base) for y = 0; half oxidized form (emeraldine base) for y = 0.5. Below: UV-vis spectra of materials in the undoped form based on disubstituted conducting polymer (PDMA and PDOA): PDMA pure conducting polymer; PDMA-MWNTs nanocomposite material; PDMA-SWNTs nanocomposite material. PDOA pure conducting polymer; PDOA-MWNTs nanocomposite material; PDOA-SWNTs nanocomposite material.

Among conducting polymers, polyaniline (PANI) and its derivatives have been deeply studied for their good electric properties, easy methods of synthesis and high environmental stability [19,20,21,22]. Scheme of the synthesis leading to the formation of a repeat unit starting from monomers is shown in Figure 3, above.

The chemistry of polyanilines is generally more complex with respect to other conducting polymers. This fact is due to their dependence on both the pH value and the oxidation states, described by three different forms known as leucoemeraldine base (LB) (fully reduced form), emeraldine base (EB) (50% oxidized form), and pernigraniline base (PB) (fully oxidized form). The most important is the EB form and its protonation by means of H+ ions generated from protic acids gives the emeraldine salt form (ES), responsible for the strong increment of conducting properties. This process is reversible and it is possible for the presence of imine groups basic sites located along the conducting polymer backbone [23,24]. The remarkable fact that the chemical–physical properties of PANI and its derivatives are pH sensitive has led to the study of these materials as sensors [17,25,26,27,28,29,30]. The doping process of polyanilines is always associated with conformational modifications of the polymer chains, due to the local distortions created by the addition of H+ ions to the basic sites and usually provides stable systems [31]. It means that the conducting polymer in the doped form can be maintained in this state for long periods of time till the material reacts with basic reagents and strongly changes its chemical–physical properties. In other words, the reversibility of the process is not spontaneous. Interestingly, fast spontaneous releasing of the doping agent from the polymer backbone was reported when substituents are present along the aromatic rings constituting the polymer backbone [25].

This method to fabricate nanocomposite materials takes into account the polymerization of monomers dissolved in a dispersion of CNTs and finds fully application in the synthesis of polyaniline and its derivatives [32,33,34,35,36]. Since the insolubility of CNTs in all common solvent, the dispersion is often obtained by means of ultrasonic equipments, taking care to maintain intact their original structure. An alternative route of synthesis is performed by using surfactants in the medium of reaction in order to ease the dispersion of CNTs. The growing polymer chains thus embed the CNTs inside the matrix by a wrapping process with no formation of strong interactions, such as covalent bonds. The nanocomposites obtained with methods of synthesis are always soluble in common organic solvents. This property is very important especially in the field of sensor devices, since their fabrication always involves the deposition of material films on suited substrates.

A further method for the fabrication of nanocomposite materials takes into account either the deposition of polymer thin films on CNTs substrates [17,37]. This method is mainly performed by previously synthesizing array of CNTs on silicon substrates. The CNTs thin film is grown by using a radio frequency pulsed plasma enhanced chemical vapor deposition (RF PECVD) system. Prior to the nanotubes growth, a Si3N4/Si substrate is patterned with platinum film (60 nm thick) by vacuum deposition through shadow masks, containing rectangular stripes 30 mm wide and a back deposited thin film platinum heater commonly used in gas sensor applications [11]. A thin film (3 nm) of Ni catalyst is deposited onto the Si3N4/Si substrates by using thermal evaporation. The CNTs film is thus obtained by pulsed RF-PECVD deposition on Si3N4/Si substrates.

UV-vis spectra of nanocomposite materials (Figure 3, below) usually show the characteristic absorption bands at about 310, 330 and 570, 630 nm due to π-π* interband transition and n-π* transition from the nonbonding nitrogen lone pair to the conduction band π*, respectively (Table 2). The presence of determined substituents along the aromatic rings is also responsible for a red shift in the transition due to the assigned to the n-π* transition from the nonbonding nitrogen lone pair to the conduction band π*.

**Table 2:** Wavelength of the peaks related to the π-π* and n-π* transitions for all materials in the undoped forms.

Material	π-π*_(Undoped)_	n-π*_(Undoped)_
PDMA	331 nm	594 nm
PDMA-MWNTs	332 nm	597 nm
PDMA-SWNTs	331 nm	597 nm
PDOA	345 nm	677 nm
PDOA-MWNTs	345 nm	685 nm
PDOA-SWNTs	348 nm	688 nm
POTO	314 nm	601 nm
POTO-MWNTs	316 nm	600 nm
POTO-SWNTs	316 nm	599 nm
POAS	328 nm	654 nm
POAS-MWNTs	328 nm	661 nm
POAS-SWNTs	327 nm	661 nm

It was in fact envisioned that electron donor substituents such as the methoxy group interacts with the surface of CNTs, while non electron donor substituents such as the methyl group have no interaction. Furthermore, the π-π* interband transition show always no shift indicating that the polymer chains of conducting are simply wrapped up around CNTs without the formation of non covalent bonds [33]. The number of substituents along the aromatic rings may also affect the formation of the polaronic states due to an increased sterical hindrance (Table 3).

If the scarcely formed polaronic states decreased the conductivity on one side, it allows a spontaneous doping process on the other. Thin phenomenon is very important since it is possible to take advantage of the spontaneous releasing of doping agent, H^+^ ions in the case of polyanilines, to set up reversible sensors for acid vapors [25]. Nanocomposites with nanotubes of other conducting polymers such as polypyrrholes can be used for gas sensing [38].

**Table 3:** Values of the band gaps (Eg _Doped_) calculated for the polaron-π* transitions of POTO, POAS, PDOA and related nanocomposites in the doped form. The calculation for PDMA and related nanocomposites was obtained by considering the π-π* transition since, for these materials, the formation of the polaronic state was impeded.

Material	Eg _(Doped)_
PDMA	3.1 eV
PDMA-MWNTs	3.1 eV
PDMA-SWNTs	3.0 eV
PDOA	2.3 eV
PDOA-MWNTs	2.3 eV
PDOA-SWNTs	2.3 eV
POTO	2.7 eV
POTO-MWNTs	2.7 eV
POTO-SWNTs	2.7 eV
POAS	2.5 eV
POAS-MWNTs	2.5 eV
POAS-SWNTs	2.5 eV

**Figure 4:**
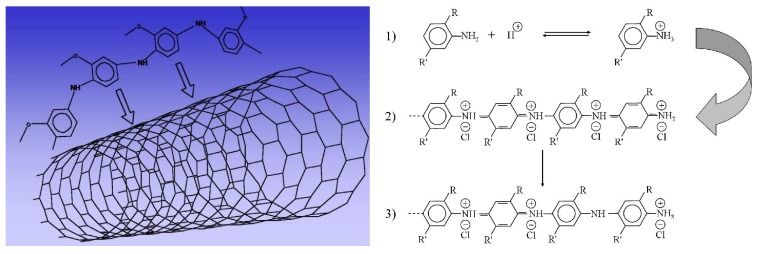
Left: Representation of nanocomposite materials based on wrapped up polymer chains around CNTs without the formation of strong interactions. Right: Scheme of the protonated pernigraniline chains growing process by addition of anilinium cations in para position. Their stability is strictly ruled by the possible delocalization of the positive charges along the polymer backbone, which is increased by the best reachable alignment (“in a plane” zigzag configuration) of the benzene/quinoid-like rings.

Experimental data related to the insertion of CNTs inside the polymer matrix (Figure 4 left) showed that the specific conductivity of the nanocomposite materials increase with respect to the pure conducting polymers. The specific conductivity is deeply affected by the formation of the polaronic state and is strictly related to the sterical hindrance of the substituents along the aromatic rings. The doping process is also responsible for the change in the morphology of the deposited films. It was envisioned that upon doping the materials deep fractures take place on the surface, thus affecting the conductivity [25].

The cyclic voltammetry measurements carried out on polyaniline and its derivatives are usually characterized by the presence of three redox couples, corresponding to the leucoemeraldine/emeraldine, intermediate emeraldine, and emeraldine/pernigraniline transitions [39]. Cyclic voltammetry of nanocomposite where conducting polymer chains have higher sterical hindrance along the aromatic rings is characterized by the presence of not well resolved oxidation peaks due to oxidized/reduced forms transitions, such as in poly(o-methylaniline)-MWNTs and poly(2,5-dimethylaniline) nanocomposite materials [17,25,26,27,28,29,30,31,40]. This behavior is not shown when the sterical hindrance decreases, thus allowing completely resolved oxidized/reduced forms transitions peaks such as in poly(O-methoxyaniline)-MWNTs nanocomposite material [41].

Nanocomposite materials based on polyaniline derivatives and CNTs were studied for their possible applications in sensor devices. The H+ doping process (Figure 4, right) is important to turn the conducting polymer into the conducting form, and it is possible to take advantage of this change in specific conductivity in order to develop sensors to determine the presence of environmental acid vapors [17,25]. Further applications of polyaniline and its derivatives in the field of sensors devices were found for the determination of for aliphatic alcohols [42], gaseous ammonia [43], nitrogen dioxide vapors [44], flammable gases such as methane [45], carbon monoxide [46], ethanol-water mixtures [47], and even humidity [48]. The various applications so far discussed, which take into account pure conducting polymers without formation of nanocomposite materials, it is important to highlight the possibility of either “tuning” the concentration of CNTs inside the polymer matrix as well as the kind and number of substituent along the aromatic rings to develop new composite materials showing improved sensing capability by means of easy method of fabrication. It is very important to underline that CNTs modified electrodes were also studied for the detection of organic molecules such as glucose [49]. In detail, a novel MWNTs-based biosensor for glucose detection were studied and compared with those of glassy carbon based biosensor. The MWNTs-based biosensor was able of a strong glucose response at a wider range of potentials with respect to glassy carbon based biosensor. Besides, the MWNTs-based biosensor highlighted a much higher stability of 86.7% of the initial activity to glucose after four-month storage, much higher than 37.2%, the corresponding value for a glassy carbon based biosensor.

Conducting polymer belonging to the class of polyanilines found also application in the field of biological molecules detection. In detail, polyaniline derivatives films, such as poly(O-methoxyaniline), doped with two different anions, specifically perchlorate and paratoluene sulphonate, electrochemically prepared on a gold substrate, were found to be improved in retaining the redox conductivity of the conducting polymer films. This behavior is similar to that of polyaniline films but unlike polyaniline films, even smaller ions like perchlorate and paratoluene sulphonate are sufficient for retaining the redox conductivity of the film, which may be primarily due to the compact, non-permeant films formed during electropolymerization. The films were found to be suitable for the immobilization of glucose oxidase enzyme, acting as an efficient sensing matrix for glucose. The films also exhibited catalytic activity towards the oxidation of nicotinamide adenine dinucleotide hydride (NADH). These studies indicate the usefulness of polyanisidine–anion films as sensing platform for biological molecules [50], adding further “tuning” effect on the sensing properties of the materials by using different doping agent from the classic proton acids. As underlined in the previous section, modified CNTs showed interesting sensing applications. These sensing properties may even range towards the field of biological applications, *i.e*., for the determination of cholosterol [51], or even microbial cells [52]. The study of these materials for a wide range of gas sensors of interest, such as carbon and nitrogen dioxide, represents now our primary objective following the early success in fabricating a spontaneous reversible sensor for acid vapors [25] and recent trends.

## 4. Langmuir-Blodgett Multilayers of Proteins for Health and Environment Sensors

**Figure 5:**
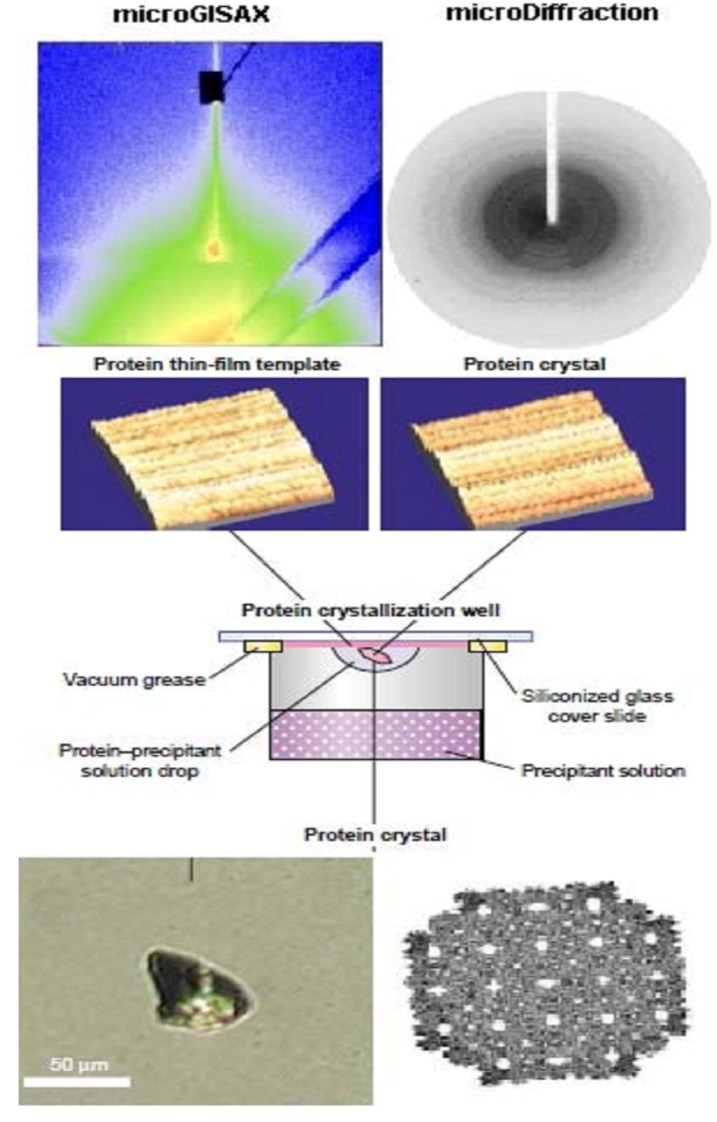
LB-based protein crystals preparation and characterization.

Here we describe protein nanostructures for device implementation, namely the LB films of lipases, laccases and metalloproteins which appear usable in biosensor applications through the formation of LB nanostructures (Figure 5). The sensors discussed herein are summarized in Table 4.

In the last decade it was shown that the LB method, consisting of the formation and proper compression of protein monolayers at the air-water interface [53], appears capable to deposit the resulting protein monolayers onto solid substrates and to preserve protein structure and function, providing new useful properties, such as protein temporal stability, film anisotropy and protein heat stability [54,55]. Some of the advantages and applications relevant to nanostructuring of the protein in the LB or LS thin films are listed below.

- The secondary structure of all studied proteins in LB films is heat-proofed up to 200 °C (for a review see [54,56]. Moreover, it was shown for LB films of photosynthetic reaction centers that special thermal treatment can improve the film ordering [57].
- The high temporal and thermal stability allow the use of these matrixes in the biocatalysts and biosensors devises. Moreover, the protein organization in the designed multiplayer can increase its enzymatic activity and the numbers of working cycles [54].
- The protein LB nanotemplate matrixes, obtained by protein monolayes immobilization can be successfully used in initiation and acceleration of crystallization of proteins, not crystallizable so far [58], introducing the new field of advanced research, namely nanocrystallography [59]. LB-based protein crystals preparation and characterization is shown in Figure 5.
- The proven crystals obtained by these matrixes have exceptional radiation stability. This can help to overcome the major problem in protein X-ray crystallography—the radiation damage issue [60].

A number of advanced methods and techniques can be used to characterize such structures:

- Nanogravimetry, namely the technique which exploits the properties of piezoelectric quartz crystals to vary their resonance frequency when a mass is adsorbed to, or desorbed from, their surface, can give information on the uniformity and reproducibility of protein LB matrix deposition. Moreover, knowing the molecular weight of the protein molecule, it is possible to evaluate experimentally the area per molecule in the film obtained, and to compare it with the area theoretically estimated for the closely packed system. The latter value can be calculated easily, knowing the geometrical parameters of the protein from the Brookhaven Protein Data Bank. In cases in which the structure of the protein has not yet been resolved, homologous protein parameters or geometrical features from molecular modeling can be used for the calculation.
- Non-contact “tapping” mode AFM can be used as a topography-sensitive method to provide the surface image of LB protein sample [61]. Moreover, this technique can be used for both proteins LB films and crystals characterization [62].
- Scattering analysis can give better in-depth insight onto the LB film long range order. Huge progress in X-ray scattering technique have been achieved, namely at ID13 beamline at European Synchrotron Facility (ESRF) in Grenoble [63], where the micro and nanoGISAX set up has been developed [64,65]. Now it is possible to investigate laterally inhomogeneous surfaces and interfaces with a two order-of-magnitude increase in spatial resolution compared to standard reflection set-ups. This allowed obtaining a coherent explanation for the origin and the molecular mechanisms of the protein heat proof in LB film. [66].
- *Ex situ* and *in situ* micro and nanoGISAX studies leaded to profound understanding of the mechanisms of the protein crystallization initiated and accelerated by protein LB nanotemplate matix [66,67].
- X-ray diffraction and powder diffraction experiment can give insight into in the protein orientation and long-range order in thin films after heating. This phenomena was studied in detail using the example of urease and penicillin G acylase [66] optimally immobilized [68,69].
- the comparative atomic structure characterization of thermophilic *versus* mesophilic proteins by X-ray crystallographic diffraction and nanogravimetric analysis in protein solution, thin film and crystal, recently pointed to the role inner bound water in determining protein thermostability ([70] and references therein).

### 4.1. Three-Dimensional Nanoporous Lysozyme Materials 

Here we describe alternative protein nanostructures for device implementation, namely protein crystals with 3D inner organization (Figure 5 below right). The construction of useful nanostructures via self-assembly of synthetic organic molecules has been successfully demonstrated for various applications, including molecular motors. The analogous use of biological molecules such as DNA or proteins, as “building blocks” for the *in vitro* construction of nanostructures is less developed and its potential still far from being fully exploited. However, protein crystals have already attracted attention as nanomaterials with promising porous and catalytic properties [71]. Self-assembly of three dimensional nanostructured functional arrays is a major challenge in nanotechnology, mainly in the fields of photonic crystals, quantum dots arrays and metallic nanoparticles. Exploitation of the inherent potential in the use of biological macromolecules—DNA and proteins—as the“guiding template” for the construction of two and three dimensional nanoarrays of nanoparticles and quantum dots is just beginning to emerge and could be exploited for a new generation of sensors.

The array of inner spacings of protein crystals routinely prepared for X-ray crystallography attracted only little attention until now: it was noted that different crystallization conditions applied to same protein may result in different packing and attempts were made to analyze protein–protein interactions for better understanding of packing patterns. The use of protein crystal spacings as a functional part for practical purposes was limited to the use of chemically crosslinked enzyme crystals as immobilized biocatalysts for a variety of synthetic applications with effective diffusion taking place via small channels. These studies clearly demonstrated that protein crystals, routinely obtained either via common crystallization procedures or by the nanobiofilm templates here described, can be readily stabilized by chemical crosslinking e.g., with glutaraldehyde. In this context it was also suggested that protein crystal spacing “channels” could be used as “natural zeolites” for chromatographic purposes.

Self-assembly of proteins into nano and microsized structures with subsequent spacing “channels” may be affected via several mechanisms:

- *In vitro* reconstitution of purified natural structural protein, e.g., reconstruction of bacterial S-layer on a supporting surface.
- Protein crystallization from saturated solutions, e.g., preparation of crystals for X-ray crystallography.
- Specific crosslinking of binding proteins by bi or multi ligands, e.g., crosslinking of lectins by di or oligosaccharides, in analogy to natural processes.
- Directed self-assembly of a binding protein into a pre-designed crystal by specific crosslinking affected by bi-ligand imposing predetermined relative orientation of the crosslinked binding protein, an approach recently introduced by our laboratory.
- Directed self-assembly of chimeric proteins into a pre-designed crystal by specific recognition of protein subunits into linear or circular structures, affected by synthetic chimeric protein “building blocks”.

The use of directed self-assembly of a binding protein via specific cross-linking into pre-designed diamond-like protein crystal and the computer simulation of the array of the enzyme crystal cavities appears particularly promising. In this case, the construction of electronic nanostructures has been successfully demonstrated, integrating in a novel “3D crystal engineering” approach protein “building block” selection (a tetrahedral lectin) and molecular modeling (for bi-ligand crosslinker design), thereby potentially providing a versatile tool for the preparation of novel composite nanostructured materials with their internal cavities as “guiding template” with potential applications in the area of photonic crystals and of semiconductors [55].

The structural characterization of the above nanomotarials stabilized and chemically crosslinked is carried out by X-ray crystallography, electron microscopy and chemical analysis. The stabilized protein crystals is typically equilibrated with solutions containing monomers, salts or metallic ions in order to yield uniform polymerization, crystallization or metallization within the cavities of the protein crystal. This is shown by the lysozyme enzyme, earlier described in [58,59] to introduce nanocrystallography and here used as model for this study: the stabilized crystals equilibrated with a series of precursors does not appear to induce any distortion in the protein template.

Lysozyme crystals were also readily stabilized by chemical crosslinking with full retention of crystal and protein three dimensional structures. All crosslinked crystals retained their shape and size, when equilibrated with a series of monomers and their combinations, all dissolved in 25% aqueous ethylene glycol, to allow for the mixing of water insoluble monomers following equilibration for 20 h at room temperature. Similarly, apart from the monomer mixture containing hydroxyethylmethacrylate, all monomer mixtures tested were readily polymerized without distortion of crystal structure, for the unique combination of acrylamide/styrene copolymerization. Finally, the resulting complete mechanical, electrical and optical properties, which are still under experimental evaluation, could lead in the future to the concrete fabrication and use of a new family of crystal protein based composite materials with exciting functional properties useful even for a new generation of sensors.

### 4.2. Lipases 

Lipases have attracted attention by their efficiency in digesting a wide range of fatty acids and triglycirides. There is a wide body of data in implementation of lipase based sensors, such as amino acid sensors [72], and also for lipid and triglycerol biosensors in medical diagnostics ([73] and references therein, [74]). Besides, the lipase, as other hydropases, induce marked local pH changes in their catalytic act which underlies viability of a lipase-based potentiometric sensor [75]. In this view it is of particular interest to consider utilization of Langmuir–Blodgett technique to develop nanostructured crystal based on interfacially activated enzymes and therefore a biosensor material.

Another protein with well-characterized nanostructured materials is Cytochromes P450 as will be discussed later. Particularly, thin films of lipase from both Mucor miehei and Candida rugosa were fabricated and characterized by UV-vis spectroscopy, Atomic force microscopy and biochemical assays [76,77]. As the first step, the M. miehei protein films were studied at the air-water interface and then transferred onto a solid support for further characterization of the enzymatic activity also *versus* surface pressure, proving that Langmuir–Blodgett film provides a better catalytic effect in lipase than a mere oil-water boundary. Moreover, improvement of lipase catalytic performance was achieved for the M. miehei *versus* the C. rugosa, despite its almost random distribution of hydrophobic patches and the low purity of its preparation. In particular, catalytic activity of lipase characterized by the maximal reaction rate found to increase over 10 times as a result of inclusion into LB films, while the substrate binding characterized by the Michaelis constant remain unchanged. Catalytic activity per mole of enzyme was found to increase with the increased number of LB layers up to five, and then decrease at 10, while the surface coverage ranged from 70% to 100% from 1 to 10 layers of lipase. This study exploits the possibility to employ LB based protein structures to use in biocatalysis, exemplified by lipase, which is known as an interfacially-activated enzyme, with olive oil as substrate, when lipase should already be in the maximally active state even without a film. We show, however, that it was possible to form even more active lipase nanostructures by the Langmuir-Blodgett technique at the air-water interface, proving that Langmuir-film provides a better catalytic effect in lipase than a mere oil-water boundary.

**Figure 6:**
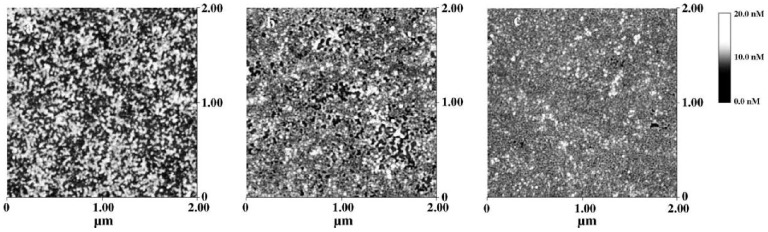

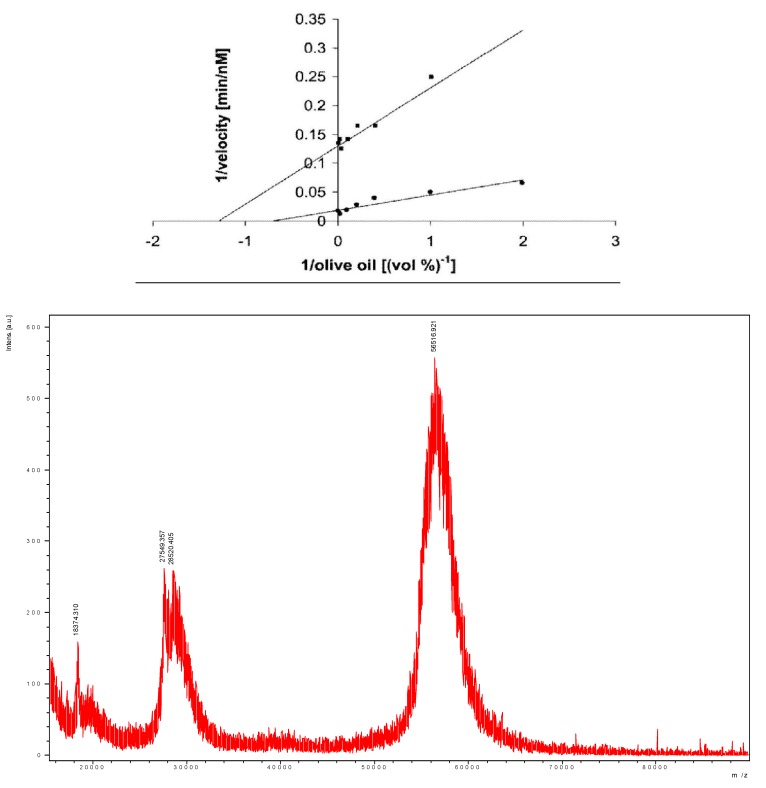
Above: AFM 2D images of immobilized lipase: (**a**) 1 layers; (**b**) 5 layers; (**c**) 10 layers of lipase. 50 µL of lipase at a concentration of 10 mg/mL was dispersed onto the air/water interface and immediately compressed. Monolayers were sequentially deposited at 20 nN/m onto a silicon slide for analysis. Center: Lineweaver–Burke plots of olive oil activity of the free (upper plot) and LB film-contained (lower plot) lipase. One LB monolayer of lipase was used for the immobilized enzyme corresponding to 10−4 mg of free enzyme. Below: MALDI TOF MS spectrum of recombinant Laccase from Rigidoporus lignosus. The spectrum was acquired in linear mode and the matrix utilized was sinapinic acid. The spectrum was calibrated externally by Protein standard solution II (Bruker). The peak at m/z = 56516 ± 94 has been assigned as a Laccase one; the other peaks at m/z = 28520 ± 83, m/z = 27549 ± 44 and m/z = 18374 ± 22 have been interpreted as different domains of Laccase erroneously expressed by the clone.

In Figure 6 above, AFM 2D images of 1, 5 and 10 lipase layers are reported. It can be observed that an increase of the number of layers corresponds to an increase of the rate of surface coverage from a value of about 70% for one layer to about 99.5% for 10 layers. It can be noted how the surface morphology of all the films is characterized by grains with a lateral size of about 200–500 nm, irrespectively of the number of layers.

Functionality of the lipase multilayers was checked in order to evaluate the effect of the LB technique on this particular enzyme, with the olive oil as substrate. The enzymatic activity of lipase was assessed by monitoring the product formation over time. The activity of lipase LB films was compared with the activity of the enzyme in absence of the film. Kinetics of hydrolytic activity of free and immobilized lipase was investigated at various concentrations of olive oil (37 °C, pH 7.7). These data were processed according to the method of Lineweaver–Burke (Figure 6, center). The apparent Km value of free lipase (0.8%, v/v) was found to be only a little lower than that of the immobilized one (1.4%, v/v). This increase in apparent Km value might be either due to structural changes in the enzyme induced by the applied immobilization procedure or due to the lower accessibility of the substrate to the active site of the immobilized enzyme. The Vmax value (55 nmol/min) for the immobilized lipase was found to be higher that of the free one (7.4 nmol/min), indicating much improved catalytic activity. MALDI TOF MS spectrum of recombinant Laccase from Rigidoporus lignosus are shown in Figure 6, below.

Thus we found the LB technique to be a suitable tool for the manipulation of lipase at the nanoscale level and studies of its interfacial activation. Application of this technique led to a better functional and structural stability of lipase which is one of the most industrially utilized enzymes, as well as providing new, albeit qualitative, insights into lipase activation mechanisms. In particular, we show that the likely key factor responsible for interfacial activation of lipase in LB monolayers is closer packing, probably acting via mechanic pressure causing the lid to open. Whether this effect could be extended to activation in organic solvent can be evaluated theoretically by calculating the volume of lipase molecule in various solvents, and the concomitant conformational rearrangements. Such studies should facilitate the choice of organic solvent or solvent mixtures allowing optimization of lipase catalysis of polymer degradation, which is impracticable in aqueous medium, and optimizing the development of nanostructured matrices for lipase use in biosensors.

### 4.3. Octopus Rhodospins

**Figure 7:**
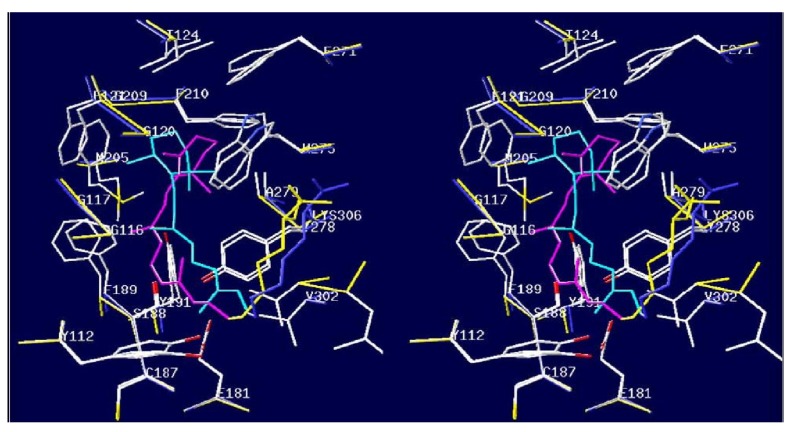

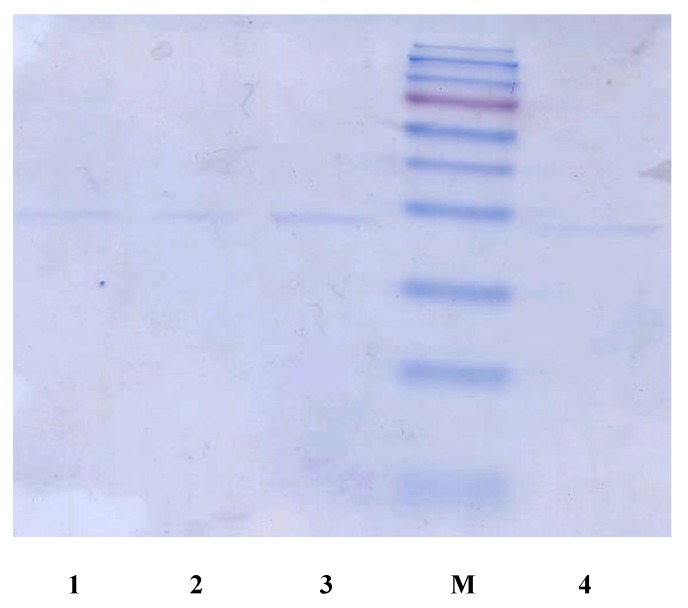
Above: Stereo view of the superimposed retinal-binding pocket for 11-cis (left) and all-trans (right) retinal. The retinal is colored in magenta and cyan respectively; backbone atoms in yellow and blue, respectively, and sidechain atoms according to the CPK scheme. Below: SDS-Page of rhodopsin protein after purification step: rhodopsin concentrated samples were run on lanes 1, 2, 3, 4 and molecular weight markers were run on lane M. Molecular weight of rhodopsin thus determined was ~33 KDa.

The visual membrane of octopus captures single photons, extremely sensitive light sensor [78]. Moreover captures polarization of light, which is the long sought solution to the problem of detectors for quantum computation detector [79]. The bacterial homolog of rhodopsins, bacteriorhodopsin, in contrast, is perfectly ordered, but does not have sufficient precision if used as a biosensor, since *in vivo* it is not a sensor but an energy transducer [80]. In [78], we compare the structure of bacteriorhodopsin (bR) with the homology model of octopus rhodopsin (octR), which is similar in topology to bR and as highly ordered in its native membranes as bR in purple membranes. Such comparison provides insights for optimization of present octR experimentation both for crystallization and for application in nanobiotechnology in a manner similar to bR, and possibly even superior in optical computation. We prove that visual membrane of octopus contains, apart from rhodopsin, a different protein which makes the membrane a natural nanocomposite. Indeed, as follows from Figure 7 above, octopus opsin should readily recombine with all-trans retinal as the superimposed structures allow one to see that octR opsin can accommodate retinal both in its native 11-cis confirmation and the all-trans conformation. This finding is supported but the experimental data [81] showing that, while both bovR and octR are easily regenerated with their native 11-cisretinal, octR can be additionally regenerated with all-trans retinal and moreover the 13-cis retinal which is the native ground-state chromophore for bR. We found that the pairwise homologies between rhodopsins from lancelet and octopus, as well as bovine and octRs, are similar (about 30% identity) but the 275th amino acid position in our octR model equivalent to the 265th position of lancelet rhodopsin is also occupied by tryptophan (data not shown). In contrast, there are no tryptophans at the corresponding position of bovR or positions close in its amino acid sequence. On one hand, this adds to the validity of our octR model since it predicts not only the experimentally known effect, *i.e*., the ability of octR to bind all-trans-retinal, but also its cause, *i.e*., the tryptophan at 275th position. On the other hand, none of the mutations proposed in this paper are at 275th position so they are unlikely to affect this property. Besides, the mutation of the neighboring Tyr278 to Trp proposed herein is unlikely to affect the conformation of Trp275 because both Tyr and Trp residues have aromatic sidechains. However, the conformations of the ionone ring in the model of all-trans reconstituted octR and the 11-cis darkstate octR are not easily interconvertible within the protein because of contacts with protein side chains. This is supported by the fact that intraprotein conversion of 11-cis to all-trans conformation in any rhodopsin requires the entire range of the photoprocess, which has the time-scale of milliseconds. Upon activation by light, followed by transition through batho- and meso- to limi-rhodopsin, the retinal molecule dissociates from octR to form the free retinal and the octopus opsin, so octR regenerates again only after the retinal has been converted into the 11-cis configuration. However, our data, together with the data of [81] as well as evidence for presence of alternative binding sites for retinal on opsin [82], suggest that, unless the all-trans retinal is quickly transformed into 11-cis retinal upon release into the lipidic matrix or bound to some transporter protein delivering it to and from the isomerization site, it will be very likely sequestered by the same octopus opsin, thus leading to the inactive form of octR thereby terminating the visual cycle. In reality, the visual cycle is not terminated, which implies that the all-trans retinal release from octR is followed by its uptake by a protein other than rhodopsin. One such protein could be the retinochrome, the retinal isomeriz ation enzyme [83]. However, the body of experimental data available for invertebrate rhodopsins suggests that the retinochrome is located away from the rhodopsin, but linked to it via a shuttle protein RALBP [84,85,86]. We will now consider the maximal distance from the site of retinal release from octR at which retinal uptake by RALBP must take place. Assume the excessively high diffusion coefficient of 10–6 cm^2^/s for retinal in the membrane, as well as the time available for the all-trans retinal before it is sequestered by the octopus opsin as 10–8 s, again unrealistically big because such process should be limited by side-chain and main-chain torsional motions of the opsin and/or torsional motions of the retinal, all of which are at the nanosecond to picosecond time-scale. Then, the maximal distance to which such ‘‘free’’ all-trans retinal can travel will be given by the Einstein–Smoluchowski equation and equal only 15 Å. Therefore the RALBP protein must be located very near the rhodopsin. Importantly, in other invertebrates like honeybee, such a retinal-binding protein has also been detected and moreover is able to perform the function of retinochrome [85]. Thus, our results suggest that another protein in addition to octR (possibly RALBP) should be located in the immediate vicinity to octR *in vivo*. In addition to stoichiometry of octR with respect to this retinal-binding protein, which should allow fast uptake of retinal released from octR *in vivo*, the visual membrane of octopus should be a natural nanocomposite containing two nanostructures protein phases. Below octopus rhodopsin is characterized for studies of its sensor properties and interactions necessary to reconstruct the bionanocomposite. Special attention is paid to purification procedures relevant to its further use in nanostructures and biosensors. Frozen eyes were hemisected, and the retinas were collected and washed with buffer A (4% NaCl, 50 mM Na-phosphate buffer pH 6.8). This procedure was carried out in dim red light [87,88,89,90]. The sample was loaded onto a sucrose cushion (40 mL 40% sucrose) and centrifuged for 60 min at 16,000 rpm in a Sorvall Centrifuge with rotor. This step was repeated three times. The sample was divided in supernatant and pellet. The four pellets achieved after centrifugations were dissolved in buffer A and they were centrifuged for 15 min at 16,000 rpm in three different times. The supernatant was removed, washed with buffer B (5 mM Tris-HCl, 1 mM DTT buffer pH 7.4) and was centrifuged for 15 min at 16,000 rpm. This step was repeated again three times.

The pellet was removed and centrifuged for 60 min at 16,000 rpm to obtain a dense mass. The sample was loaded onto a 200 microm GTP, shaked and centrifuged for 15 min at 16,000 rpm. This step was repeated again three times. SDS-PAGE was performed according to the method of Laemmli [91,92]. Rhodopsin was resolved on 15% SDS/ PAGE gels (10 cm long, 1 mm thick). Gels were stained with 0.1% Coomassie brilliant blue R-250 in 45% methanol and 10% acetic acid and properly destained. The rhodopsin obtained from Octopus vulgaris was purified by FPLC (DEAE column 4.5 mL volume, 0.77 × 10 cm dimensions, 0.8–5 mL/min flow, 3 bar pressure). The elution of the proteins was performed in 20 mM Tris-HCl, 0,05% DDM, 1mM DTT, 1M NaCl buffer pH 7.4. Each 1 mL of the 30 fractions collected was eluted with a flow of 1 mL/min. The rhodopsin particles were eluted as a single peak. The fractions with this peak were concentrated for 30 min. The SDS-PAGE of the fraction at this peak revealed a purified band to ~33 KDa (Figure 7, below) in complete agreement with the data of [93,94]. Time ago sensors were built either for anesthetic vapor based on bacteriorodhopsin [95] or for routine contaminants testing based on DNA [96] and now our goal is to construct a more effective sensor based on the results so far obtained and those still pending on the Octapus Rodhopsin.

### 4.4. Laccases

Matrix assisted laser desorption ionization mass spectrometry is usually employed to characterize bimolecules. Spectra analysis can also provide fundamental information about the state of degradation of a protein, for example after the immobilization on the surface of the biosensor.

We analyzed different proteins interesting as sensible elements for the realization of a biosensor, some of which purified in our laboratories, including laccase from Rigidoporus lignosus (Figure 6 below), bovine Cytochrome P450scc and initiation factor 2 alpha.

Through these measures we evidenced, in particular, some transcriptional errors occurred during production and purification of high weight proteins. It is known, in fact, that expressing foreign proteins at high rates can lead to nutritional stresses in the production bacteria. Such stresses markedly increase the frequency of translational errors [97]. The extent of the bacteria stress response is determined by the specific properties of the recombinant protein, and by the rates of transcription and translation [98]. In recombinant Laccase solutions, expression was detected, together the whole protein, of some single domains. The acquired spectra in Figure 6 below can be utilized as control to estimate the degradation of the proteins once immobilized.

### 4.5. Cytochromes

One of the most interesting metalloprotein is Cytochrome P450 which represents a numerous family of heme-containing monooxygenase enzymes. While in prokaryotes cytochromes P450 usually serve a function of synthesizing construction material, in eukaryotes their functions are metabolizing a diverse range of organic sybstrates. Thus, mammalian cytochromes P450 are components of membranes and are involved in biosynthesis and metabolism of many physiologically active substances; moreover, these cytochromes are unique in their ability to catalyze metabolism of xenobiotics, substances of an origin foreign to the organism such as drugs, toxins, environmental pollutant. Since expression of new cytochromes P450 in mammals is affected by various factors such as diet an environment, it can be envisaged that by means of genetic engineering, a cytochrome p450 can be designed to capture, and a P450-based sensor developed for almost any organic substance. Importantly, either optical or electrical sensor is feasible using cytochromes because substrate binding causes both changes in heme visible spectrum, and produces an electrical signal.

**Figure 8:**
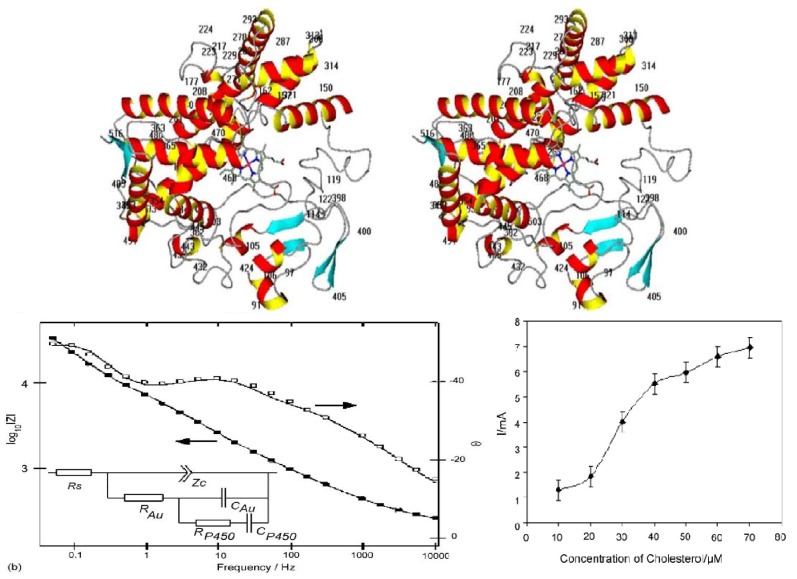
Above: Stereo view of homology model of Cytochrome P450scc, based on Cytochrome 450 2B4. Acidic amino residues are labeled. Secondary structure elements are colored. Heme is shown as sticks. Center: The impedance characteristic for an Au–P450scc electrode in 100 mM phosphate buffer, 50 mM KCl, pH 7.4. The lines show the best fit to the experimental data. The insets present the equivalent circuits fitting the impedance data. RS is the uncompensated solution resistance; ZC is the double layer capacitance; RAu and CAu are the ohmic and capacitive contributions respectively of the gold nanoparticles on the electrode to the impedance, while RP450 and CP450 are the same contributions respectively to the impedance due to presence of cytochrome P450scc on the electrode. Both plots represent the phase (□) and the modulus (■) *vs.* frequency. Below: The amperometric response of screen-printed rhodium–graphite Au–P450scc electrode to increasing cholesterol concentration. About 100 µM stock solution in 0.3% sodium cholate, measured when fractions of 10 µM of cholesterol has been repeatedly added. The total volume of electrolyte was 60 µL. The current was measured at the potential−400 mV (*vs.* Ag/AgCl).

One example is the amperometric sensor for which we predicted electrochemical properties by molecular modeling. Particularly, we built a homology model of Cytochrome P450scc based on the structure of Cytochrome P450 2B4 (Figure 8 above), with special focus on the electrochemical implications of the obtained structure, and comparing it to predictions following from homology models based on Cytochrome P450cam and P450 2C5. The strategy we adopted for identifying mutations capable of altering th e redox potential of Cytochrome P450scc is based on the fact that the redox potential of the native Cytochrome P450scc is very low, 412 mV [99], and is shifted to about 250 mV upon binding of cholesterol [100]. This is just sufficient for electron transfer from adrenodoxin, which has the redox potential of 276 mV [101]. We decided to explore the possibility of mutating the negatively-charged residues for two reasons. First, isosterical mutations are available for such residues, namely Glu-Gln and Asp-Asn, allowing neutralization of the charges, with the minimal chance of disturbing the overall structure.

This, of course, will allow shifting the potential only in the positive direction. However, that is exactly what is necessary for improving electron transfer. Besides, shifting of Cytochrome P450scc redox potential to negative values can be readily done by chemical modification as follows from [102] where Cytochrome P450scc was transformed into a semisynthetic flavocytochrome. We assumed that the redox potential shift upon such mutation is mediated by electrostatic field of the protein, and exploited the superposition principle valid for electric fields. Particularly, the electrostatic interaction energy between the heme and the amino acid charges can be expressed by product of potential from the heme iron and the charges of the amino acid residues. We used only the charge of heme iron in the electric potential calculations instead of the distribution of partial charges over the entire heme.

Calculations allowed us to predict large shift of the potential for residues Glu468 and Glu470 of Cytochrome P450scc. These residues are located in the immediate vicinity of the heme, and belong to the heme-binding I helix. In our model, Glu468 is completely water-exposed, while Glu470 is buried, but this is compensated by hydrogen bonding of the O-gamma atom of Ser209 and the N atom of Ala467 with the Glu470 carboxyl. Besides, it is located near the heme, so it can be accessible to water through the same channel as the heme. The implication of these studies for optimal sensor construction is linked to the outcome of electrochemical evaluation of the new mutant cytochrome being produced by the above suggested site directed mutagenesis.

Towards implementation of an amperometric sensor, investigation of electron transfer between cytochrome P450scc (CYP11A1) and gold nanoparticles immobilized on rhodium–graphite electrodes was performed [8]. Thin films of gold nanoparticles were deposited onto the rhodium-graphite electrodes by drop casting. Cytochrome P450scc was deposited onto both gold nanoparticle modified and bare rhodium-graphite electrodes. Cyclic voltammetry indicated enhanced activity of the enzyme at the gold nanoparticle modified surface. The role of the nanoparticles in mediating electron transfer to the cytochrome P450scc was verified using ac impedance spectroscopy (Figure 8, center). Equivalent circuit analysis of the impedance spectra was performed and the values of the individual components estimated. On addition of aliquots of cholesterol to the electrolyte bioelectrocatalytic reduction currents were obtained (Figure 8, below). The sensitivity of the nanoparticle modified biosensor to cholesterol was 0.13−1 µAµM in a detection range between 10 and 70 µM of cholesterol. This confirms that gold nanoparticles enhance electron transfer to the P450scc when present on the rhodium-graphite electrodes.

Cytochrome P450scc was found to be suitable for nanostructure assembly by LB technique combined with site directed mutagenesis [103,104,105]. Molecular modeling and protein engineering were synergically employed to improve the fabrication of cytochrome P450scc mutant nanostructures for biodevice assembly. The optimization of protein three-dimensional structure by molecular modeling was performed to predict a P450scc mutant which could improve the process of molecules’ immobilization onto solid supports. Engineered cytochrome P450scc thin films were prepared and characterized by various biophysical techniques such as π–A isotherms, surface potential measurements, Brewster angle microscopy, UV-vis spectroscopy, circular dichroism, nanogravimetry, and electrochemical analysis. We show that biomolecules modified by protein engineering that represent a new and powerful approach for obtaining synthetic simpler artificial structures with new or improved properties (*i.e*., specificity, stability, sensitivity, *etc.*) useful for biosensors development.

### 4.6. Bacterial Globins

Unlike animal globins which typically function as carriers and/or storage agents for oxygen, the function of bacterial globins is that of oxygen sensors. Namely, while animal globins capture or release oxygen *in response to* external signals such as change of local pH and binding to other proteins or membranes, the mode of operation of bacterial globins is inverse: they *generate* a signal in response to binding of oxygen. For example, this is typical of aerotaxic bacteria that need to move as close as possible to the source of oxygen for their living. Thus, bacterial globins are ready made oxygen sensing elements. Generally, the signal they generate consists in several angstrom scale motion of the C terminus of the protein that in principle can be detected optically by attaching fluorescent labels and thus implementing a sensor. However, oxygen binding to heme proteins is known to affect electrochemical potential of the latter, and thus can be detected amperometrically, similarly to the sensor based on other heme proteins, cytochromes P450 (Table 4).

**Table 4:** Examples of sensors originating from protein Langmuir-Blodgett technology.

Protein and species	Sensing element/analyte	Transducer
LIPASES [76,77] Mucor miehei, Candida rugosa	Active site/Ttriglycerides, fatty acids	Release of protons -> Change of local pH* -> voltage
OCTOPUS RHODOSPIN [78,79,87]	Retinal/Light, including single- photon and polarization	Conformational/nanoscale motions*
BACTERIORHODOPSIN [95] Halobacterium Halobium	Overall conformation of protein/Anaesthetics	Pumping of protons -> voltage
CYTOCHROMES P450 [8,103,104,105] Multiorganism P450scc (mammalian adrenal cortex), P4501A2 (mammalian liver), P450cam (bacterial)	Heme/Multisensors. Almost any xenobiotic/organic molecule will induce expression of its appropriate Cytochrome P450	Change in electrochemical potential of heme -> voltage/current
LACCASE Rigidoporus lignosus, Trametes versicolor	Active site/phenolic compounds	Oxygen consumption* -> current
BACTERIALHEMOGLOBIN Methylacidiphilum infernorum	Heme/Oxygen (reversible), cyanide, carbon monoxide (irreversible)	Conformational/nanoscalemotions*; Change in optical and electrochemical properties

* Can be converted to optical signal by attaching fluorescent labels.

## 5. Nucleic Acid Programmable Protein Arrays for Nanogravimetric Sensor

Protein Microarrays represent an innovative and versatile approach to study protein structure abundance and function at an unprecedented scale. There has been a marked increase in the use of this technique to map proteins and their interactions with various other molecules, and to identify potential disease biomarkers, especially in the area of cancer biology and cell cycle progression. Advances in genomics and proteomics have created a demand for miniaturize robot platforms for the high throughput (HT) study of proteins on a solid surface at high spatial density. Protein microarrays can be considered for use as biosensors because they consist of immobilized biomolecules spatially addressed on substrates such as planar surfaces (typically coated microscope glass slides), microwells or arrays of beads. Immobilized biomolecules, here referred as probes, usually include oligonucleotides, PCR products, proteins, peptides, carbohydrates and other small molecules. Ideally, probes should retain activity, remain stable and not desorb during the experimental steps. The collection of molecules arranged (in arrays) on the substrate is then probed with cellular extracts, serum, PCR products or other samples chasing for molecular recognition events [106,107]. In spite of the problems related to the manufacture and use of microarray technology for proteins [108], a variety of interesting applications of this technique have appeared in the literature in the last years as reported by several recent reviews [106,109,110,111,112]. A challenging aspect in protein microarray technology development is the difficulty in maintaining the native state of the protein upon surface immobilization. Moreover, as many proteins are labile, the overall process in a microarray experiment (expression, purification, spotting and microarray storage) must be planned and carried out in order to maintain protein integrity [113]. A useful strategy in this regard has been proposed by Ramachandran in 2004 [114]. The authors of this work spotted protein expression plasmids instead of purified proteins on the microarray surface generating a Nucleic Acid Programmable Protein array (NAPPA) which reduces the process of building protein microarray to a single step [106]. Current detection strategies for protein microarrays are generally classified as [115]: Label-free methods, including mass spectrometry (MS), surface plasmon resonance (SPR), atomic force microscopy (AFM), micro-electromechanical systems (MEMS) cantilevers and quartz-crystal microbalance analysis (QCM), and labeled probe methods, including fluorescence, chemiluminescence, electrochemiluminescence and radioactivity detection. Although antibody-based methods of protein detection are sensitive and simple, high quality antibodies do not exist for every protein of interest and antibody development is both time-consuming and expensive. Label-free methods and Mass Spectrometry in particular offer a powerful and general way to circumvent the need for specific antibodies for each protein of interest. Mass Spectrometry, moreover, has the unique advantage of being able to determine not only the presence but also the identity of a given ligand. The difficulty in obtaining purified proteins has no doubt limited the number of studies using functional protein microarrays. A completely different strategy avoids the need to purify proteins in advance and instead relies on the production of proteins on the microarray surface using a cell-free transcription/translation reagent. The recent development of a self-assembling protein microarray, called NAPPA is based on this principle [116]. Building upon the successful use of *in vitro* translated protein in standard scale applications; Ramachandran *et al* substituted the use of purified proteins with the use of cDNAs encoding the target proteins at each feature of the microarray. The proteins are transcribed and translated by a cell-free system and immobilized *in situ* by means of epitope tags fused to the proteins. This approach eliminates the need to express and purify proteins separately and produces proteins at the time of the assay, abrogating concerns about protein stability during storage [114]. Through testing a variety of cDNA printing schemes, Ramachandran *et al* found that an optimum balance was required between binding DNA efficiently and maintaining a DNA conformation that supported efficient transcription and translation. The most efficient strategy coupled a psoralen-biotin conjugate to the expression plasmid DNA with the use of ultraviolet light, which was then captured on the surface by avidin. The addition of a C-terminal glutathione S-transferase (GST) tag to each protein enabled its capture to the array through an antibody to GST printed simultaneously with the expression plasmid [114].

To activate and use the array, a cell-free coupled transcription and translation system (such as reticulocyte lysate containing T7 polymerase) was added as a single continuous layer (not discrete spots) covering the arrayed cDNAs on the microscope slide. To test the system, expression plasmids encoding eight genes were immobilized onto an array at a density of 512 spots per slide. Expression of target protein was confirmed with an antibody to GST (different from the capture GST antibody), and the signals were measured with a standard glass slide DNA-microarray scanner [114]. This approach produces a sizable amount of protein per feature (270–2700 pg), averaging about 10 fmols. As the proteins are freshly synthesized just-in-time for assaying, there is less concern about protein stability. The microarrays are stable dry at room temperature until they are activated to make protein. This approach has been optimized for the detection of protein-protein interactions and for the co-expression of both the target and query proteins, eliminating the need for any purified proteins. In a protein interaction mapping experiment recently reported by us among 30 human DNA replication proteins, 85% of the previously biochemically verified interactions were recapitulated. NAPPA is well suited to the detection of protein-protein interactions because both the target proteins (bound to the array) and the query protein (used to probe the array) can be transcribed and translated in the same extract.

The key requirements for any new label-free protein microarray sensing technology are that it should be compatible with high throughput, HT, (multiplexed detection) methods, should be able to detect small molecules binding to immobilized protein targets, should be able to detect interactions involving biomolecules present at low concentrations in the sample, and have a wide dynamic range. The performance of a sensing technology is often characterized by sensitivity, resolution, and detection limit. In the case of fluorescence detection and protein arrays, the measured parameter is fluorescence intensity and the parameter to be determined is the number of molecules bound to the immobilized protein. Resolution is the smallest change above the noise floor of the detector in the measured parameter that can be reliably detected. These are critical parameters because their values indicate the feasibility of using a technology for a specific experiment. For example, when studying small molecule interactions with immobilized proteins, the resolution governs the smallest molecular weight for the small molecules that can be studied. Sensitivity and detection limit will govern the lowest concentration of an analyte that can be detected [113].

All label-free detection methods are promising tools to characterize binding events on surfaces (Fuentes *et al*., in preparation; Sartore *et al.*; In preparation; Adami *et al.*, In preparation). They do not require labeling of molecules that may affect protein activity. Coupling protein microarrays to real-time and label-free detection systems compatible with high-throughput methods would strongly enhance the ability to understand protein function on a proteome scale.

Matrix-assisted laser desorption ionization mass spectrometry (MALDI-MS) has quickly developed into one of the principal means for the mass spectrometric characterization of biological macromolecules. Some notable features of this technique are a sensitivity in the femtomole range, an accessible mass range with an upper mass limit approaching 300 kDa and the capability for analyzing mixtures. There is considerable interest in the direct mass spectrometric analysis of biological samples that have been immobilized on different kinds of surface [117] and moreover it appears particularly suitable since it permits to identify via fingerprinting the molecula adsorbed.

**Figure 9:**
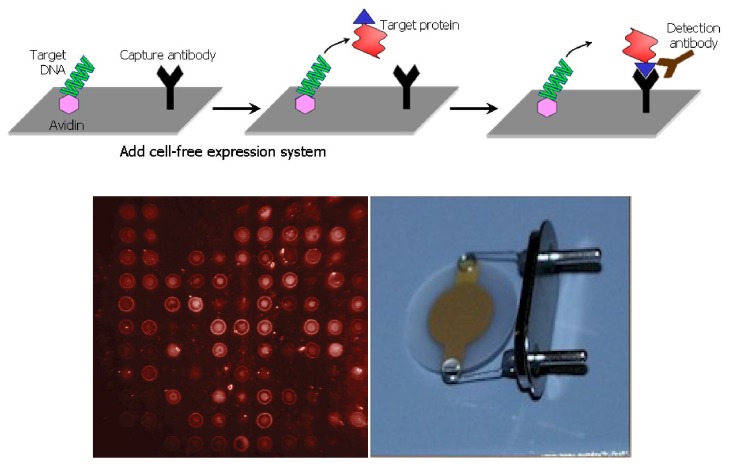
Above: NAPPA technology [103]. Below left: NAPPA region image with human kinase proteins acquired by the DNASER device. Below right: Multi-nanogravimetry quartz crystals for biosensor will consist of four gold coated quartz crystals 8 mm wide on which will be spotted NAPPA genes in the geometry previously adopted, *i.e*., 16 spots (4 × 4)—each of 300 microns of diameter—spaced of 750 microns, center to center.

In NAPPA technology (Figure 9 above), full length cDNA molecules are immobilized on a microarray surface and expressed *in situ* using a mammalian cell-free expression system (rabbit reticulocyte lysate). A fusion tag present on the protein C-terminal is recognized by a capture molecule arrayed (along with the cDNA) on the chip surface. This capture reaction then immobilizes the protein on the surface in a microarrayed format-as shown below—with a similar orientation with respect to the matrices. In a first fluorescence-based study we simultaneously express multiple proteins to build multiprotein complexes as well as map binding domains among interacting partners involved in the lymphocytes process, further enhancing NAPPA’s robustness, reproducibility, and utility as a protein microarray. An example for human kinase proteins is shown in Figure 9 below.

We are implementing a procedure to analyze Nucleic Acid Programmable Protein Array (NAPPA) [113,114,118,119] by MALDI TOF mass spectrometry [107] on a metal surface.

We performed different tests to optimize the preparation of the microarray surface , the matrix for the protein bound on the array to allow its desorption and ionization, and the placing of the matrix on the array in order to maximize the sensibility of the measurement. Mass Spectrometry in conjunction with bioinformatics has recently proven capable, after digestion of the proteins synthesized on the array of performing fingerprints and identification [113]. We performed protein expression then proceeded with the fingerprint of the proteins synthesized (p53, jun, CdK2, CdKN1A). To eventually identify the proteins captured by the MALDI-TOF technique, the mass lists were submitted to a data bank search using Biotools (Bruker Daltonics, Leipzig, Germany) software in order to obtain their fingerprint [120].

Based NAPPA a nanogravimetric sensor is being developed utilizing this powerful technique to monitor adsorption processes of different biomolecules such as DNA, proteins, lipids, and antibodies including functionalized surfaces. The quartz crystal microbalance (QCM) utilized is a Nanogravimetry device that exploits acoustic waves generated by oscillating piezoelectric single crystal quartz plate to measure frequency 1: at certain frequencies, the amplitude of the oscillation driven by AC field will be strongly enhanced. The crystal will be in resonance at this characteristic frequency. The resonance frequency decreases at mass increasing, provided it is firmly attached to the quartz crystal surface. By monitoring the frequency in real time, the temporal change in mass on the crystal can be investigated in a label free fashion.

Combination between conventional quartz crystal microbalance and dissipation D-factor can be used as a fingerprint to characterize molecular interaction and adsorption of different biomolecules.

In our earlier study we pointed out that it is possible to apply this label-free technology to NAPPA technology and now we employ this technology to realize a new innovative kind of biosensor.

For each of the four distinct quartz crystals, each box will consist of the same gene. For this first step of the study we will generate a biosensor employing three different genes plus one NAPPA gene-free as control. In particular the genes to be spotted solely for health are:

- Cytochrome P4501A2 (**Cyp1a2** in HIP Plasmid ID Database) for clozapine sensing.
- Cytochrome P450scc or P45011a1 (**CYP11A1** in HIP and DNASU Plasmid ID Database) for LDL cholesterol sensing (P450 E468Q).
- Laccase (find the gene in HIP Plasmid ID database) for health relevant compound.

In particular the three genes to be spotted for health or environment are:

- Lipase (**lpl** in DNASU database) for clozapine sensing.
- Cytochrome P450 2B4 for styrene sensing.
- Laccase for phenole derivative sensing.

Design and properties expected from NAPPA-based sensors are being defined with respect to built biosensors in standard amperometry-conduttimetry configurations using LB protein layers, gold nanoparticles and Anodic Porous Allumina.

As specific control for a quantitative assay background we use a gene-free NAPPA.

## 6. Conclusions and Perspective

Nanoscopic control of composites, including nanostructuring of biological objects, has opened a unique opportunity to create a wide range of sensors, highly optimized from the molecular scale up. For example, carbon nanotubes, for use as gas sensors as specified in Section 3, were recently complemented by another carbon based nanostructured material, graphene [121]. The ability of organic Langmuir monolayers was expanded to capture the atomic-scale details of nucleotides by mechanical fine-tuning [122,123], thus creating organic sensors for biomolecules with specificities close to those of biosensors. With respect to biopolymers especially proteins, this opportunity as discussed above may evolve into an opportunity to routinely develop biosensors for practically any organic substance. Cytochromes P450 that are able to metabolize a wide range of organic substances are especially relevant. However, biosensor development is limited by the set of materials available to the developer at a given moment, such as the protein or the nanocomposite matrix. Then, more often as not, the sensor developer asks the question “what sensor can I create with the given protein and the given matrix?”, and there is no guarantee that, however successfully the biosensor is optimized, the optimization is performed within the predefined choice. Since the choice is made by (a team of) humans, it is invariably to some extent arbitrary. To avoid this, the question should be reversed, namely “what protein and what nanocomposite matrix could be optimal for the sensor for the given substance and the given immobilization conditions”? The trivial example is the enzymes, which exist in numbers of splice isoforms, different in functional activities and immobilization properties. Moreover, such differences are often typical for different commercial preparations of the same protein. The solution seems to be a database (in progress) of protein–nanostructure interactions which would allow, not only selection of a nanostructure for the given protein and/or the given biosensor task, but also to optimally select all the components of the biosensor from the sensing protein up to biosensor hardware, starting from the given range of substances and the operating conditions of the sensor.
